# Supplementary material for: pVACtools v6: A comprehensive suite for neoantigen prediction, visualization, and therapy design
Source: ArXiv. 2026 Jun 25:arXiv:2606.26659v1. Preprint. [Version 1] (PMC13321332)
Supplement: Supplement 1 [file NIHPP2606.26659v1-supplement-1.pdf]

Supplementary Figures

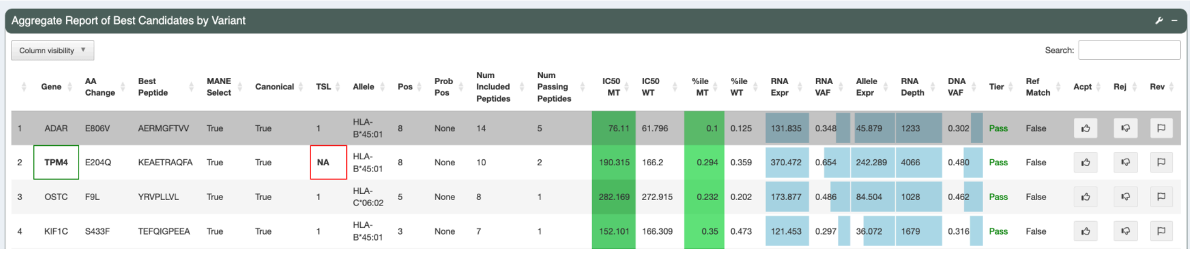

Supplementary Figure 1. **The pVACview main module interface, which summarizes data from the pVACseq aggregate report and other pVACseq outputs in an interactive manner**. Each row shows one variant, with the proposed representative candidate (Best Peptide), along with its Tier. Selecting each row allows users to explore extensive further details on the top prioritized peptide for each somatic variant, additional ranked peptides, transcript annotation information, individual algorithm predictions, etc.

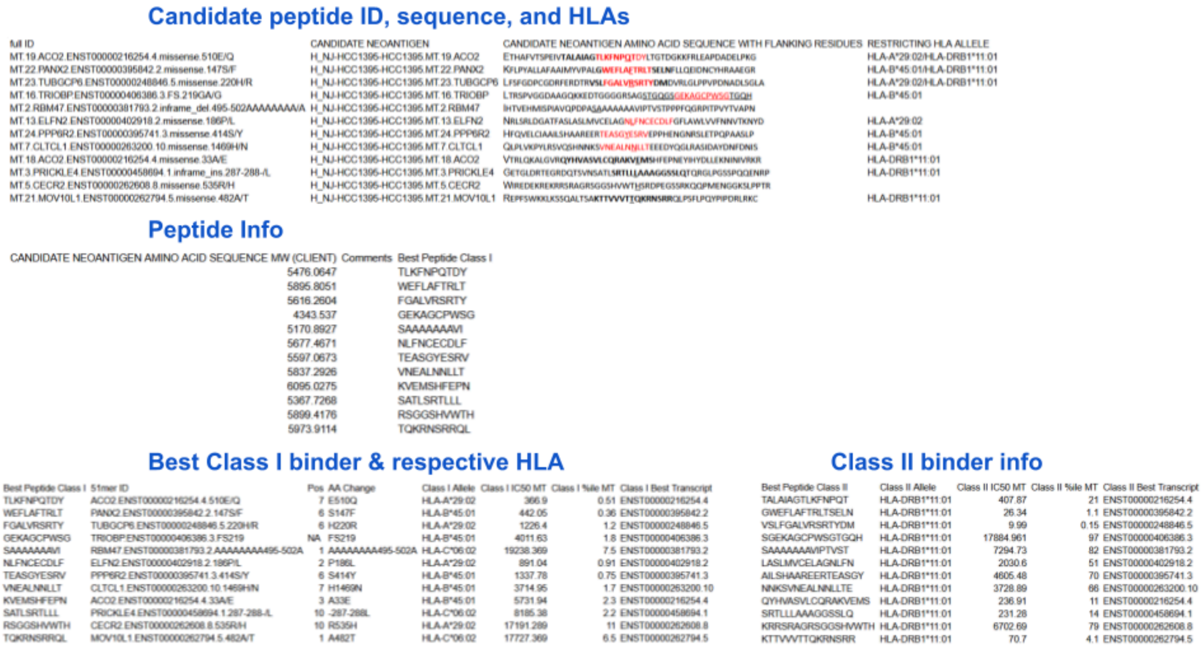

Supplementary Figure 2. Example of color-coded excel peptide sheet from ‘pvacseq create_peptide_ordering_form’. Figure shows an excel sheet with mutated sequence containing 25 flanking amino acids on either side, with mutated amino acid(s) underlined, representative MHC class I binder bolded, representative MHC class II binder colored in red, along with best class I/II binders-HLA information and the corresponding molecular weight. User-defined problematic amino acids (eg. Cysteine) are printed in larger font.

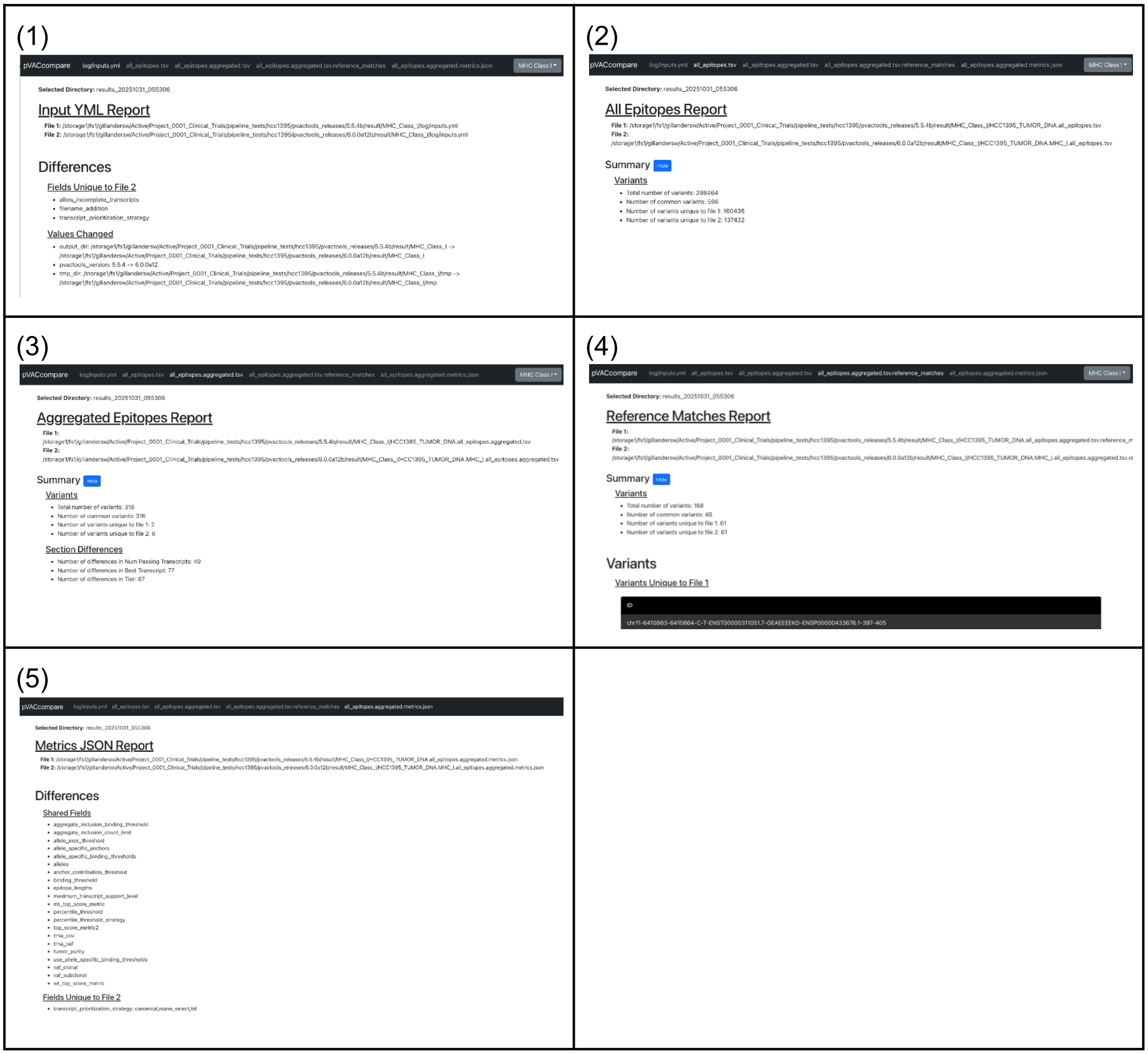

Supplementary Figure 3. pVACcompare has 5 output files.
(1) log/inputs.yml (which specifies differences in input file and pVAC run set up);
(2) all_epitopes.tsv (summarizes total common and unique variants);
(3) all_epitopes.aggregated.tsv (difference in variants, best representative transcript and candidate Tier);
(4) all_epitopes.aggregated.tsv.reference_matches (summarizes count and identify of variants with reference proteome match across runs);
(5) all_epitopes.aggregated.metrics.json (summarizes the neoantigen metrics, or setup in each run, facilitating comparison of runs from different pVAC versions).

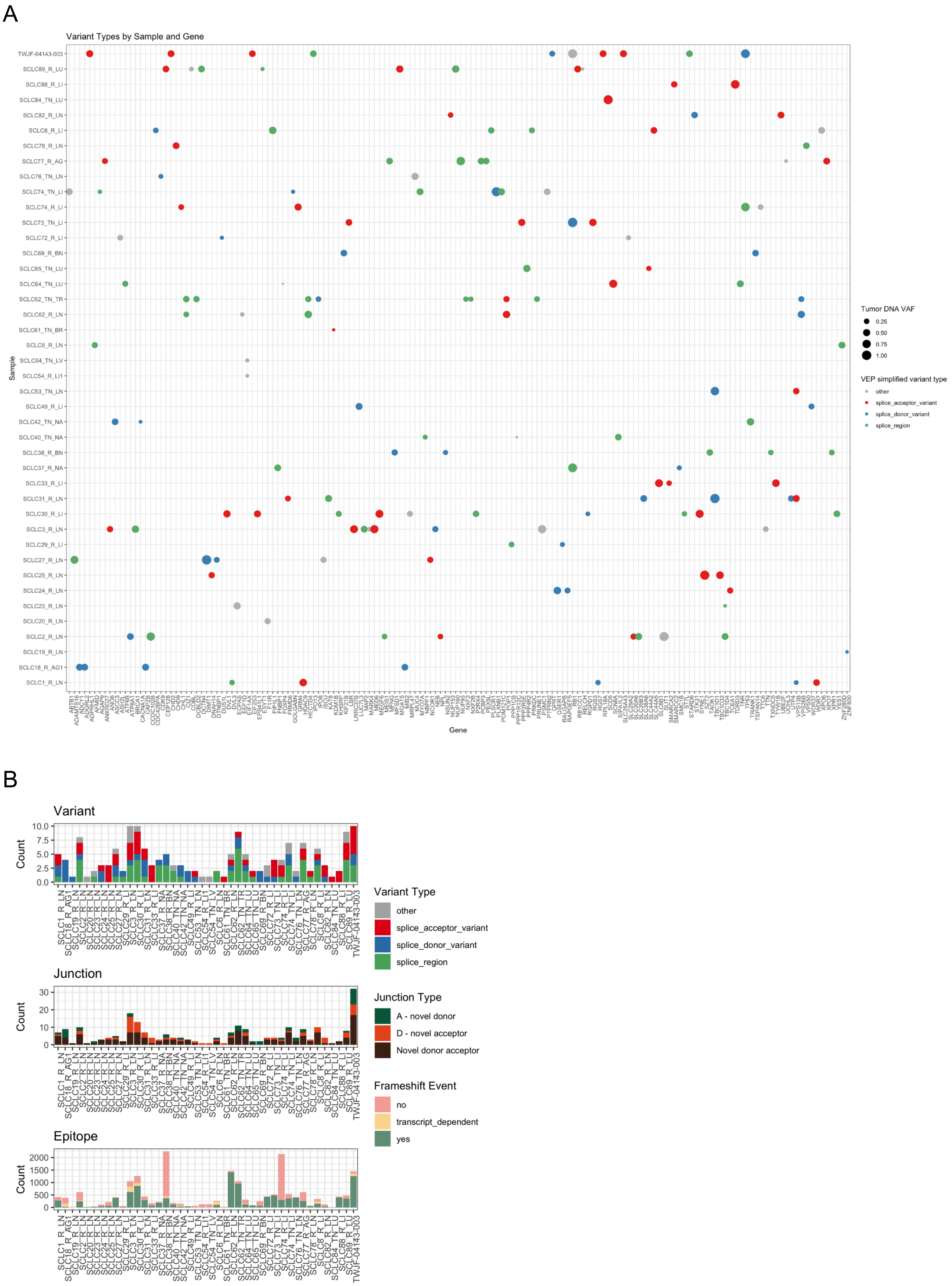
A
Variant Types by Sample and Gene
Sample
Gene
Tumor DNA VAF
0.25
0.50
0.75
1.00
VEP simplified variant type
other
splice_acceptor_variant
splice_donor_variant
splice_region
B
Variant
Count
Variant Type
other
splice_acceptor_variant
splice_donor_variant
splice_region
Junction
Count
Junction Type
A - novel donor
D - novel acceptor
Novel donor acceptor
Epitope
Count
Frameshift Event
no
transcript_dependent
yes

Supplementary Figure 4. **pVACsplice predicts multiple cis-splicing variants in SCLC cohort**. **(A)** Dot plot summarizes the cis-splicing variants observed across the cohort. Dots are sized according to variant tumor DNA VAF, and colored based on VEP annotation. Putative neoantigens are identified for key drivers TP53 and RB1 - the two most frequently altered tumor suppressor genes in SCLC.**(B)**: Summary of pVACsplice outputs including splice variants, junctions, and predicted MHC binders across the cohort. Variant counts in this panel refer to the number of variants passing the pVACsplice default filters. Junction counts illustrated here are for junctions associated with these variants that are confirmed as absent from normal tissues in GTEx.

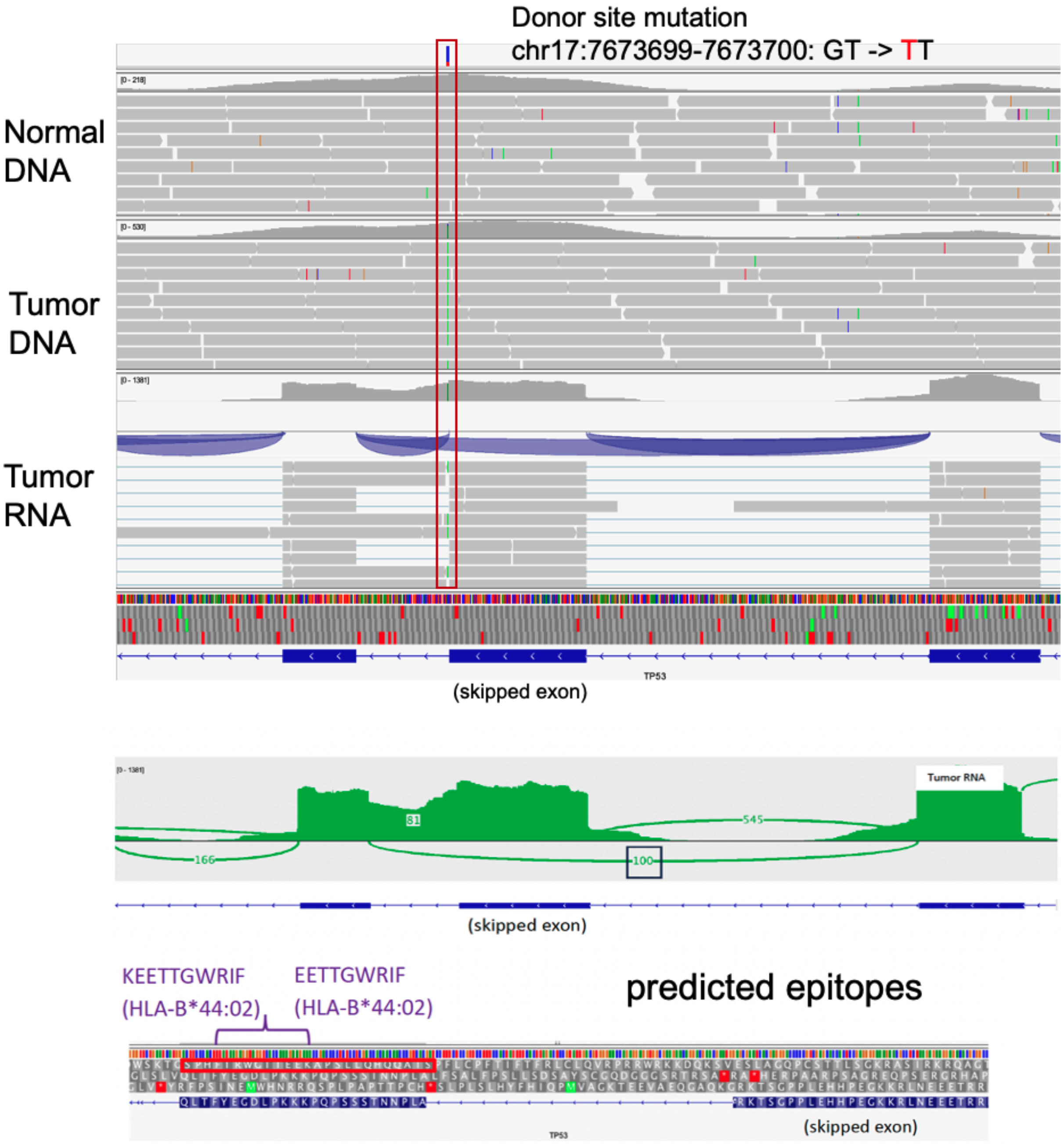

Supplementary Figure 5. Illustrative example: a mutation at the splicing donor site causes an exon skipping event and creates multiple potential epitopes as predicted by pVACsplice. The donor site mutation is tumor-specific, disrupting the canonical splicing donor dinucleotide GT, causing skipping of exon 4, thereby creating a novel exon3-exon5 junction that is robustly supported (with 100 reads assigned to the novel junction). This event is predicted to produce a frameshift and shortened TP53 protein. The mutant protein sequence contains predicted strong MHC binders for the patient's HLA allele.

ETVMPSALVRGPLQPVSLLACPFRHRC
GPGPG
RHEMVAKPPAMCSRFAKDLRPEQYIKNSFQ
GGS
GYIRQVGDFHQVIIRGRGHILPYDQPLRAFDMI
GGS
ALEREEEEERERARLWERRQQRKNREAFQTF
HHHH
PNQTVVKMFLVTFDFSNMPAAHMTFLRHRLFL
GGS
SGGGPGAQHSAMPAKSKEELSEGSRKKKT
GGS
CEVGMAYSMEKYRRTDNMARVMVRYMKY

pvacV4_IC1500_Clip5

SCEVGMAYSMEKYRRTDNMARVMVRYMKYLRQK
GGS
ALEREEEEERERARLWERRQQRKNREAFQTF
NETVMPSALVRGPLQPVSLLACPFRHRC
GPGPG
GYIRQVGDFHQVIIRGRGHILPYDQPLRAFDMI
GGS
NMKRHEMVAKPPAMCSRFAKDLRPEQYIKNSFQ
HHHH
PNQTVVKMFLVTFDFSNMPAAHMTFLRHRLFLV
GGS
SGGGPGAQHSAMPAKSKEELSEGSRKKKTKKAD

pvacV5_IC1500_Clip5

Supplementary Figure 6. Vector designs for test case G104 under the IC1500_Clip5 setting, shown side-by-side for pVACvector version 4 and version 5. Version 5 produces a solution that fully preserves the binding core (GSRKKKTKK). Binding cores are colored red. Locations of binding core with clipping event of interest are highlighted in yellow.

A

Motif from experiment

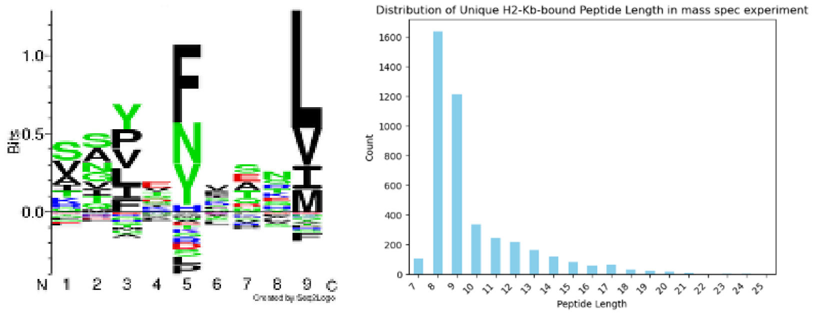

Motif from reference database

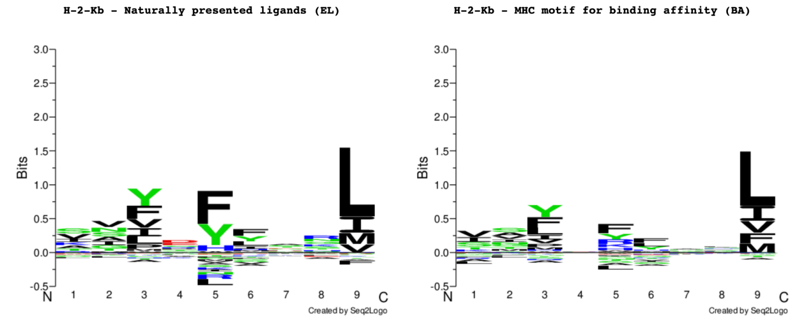

B

Motif from experiment

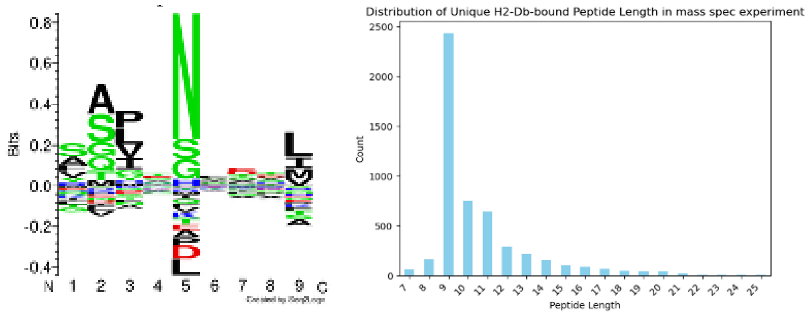

Motif from reference database

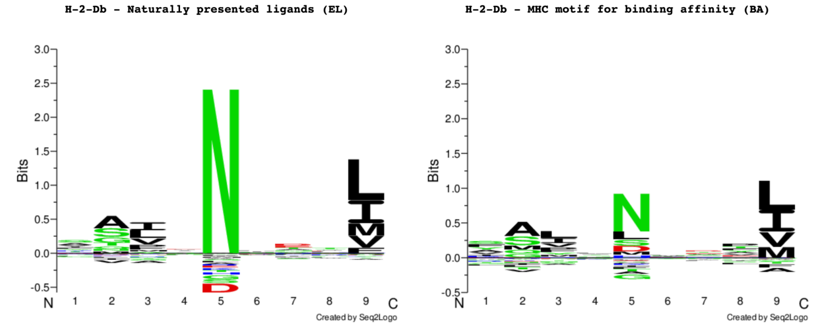

Suppl Fig 7. Mass-spec identified MHC binders from experiment have sequence motifs similar to motifs from reference database NetH2pan. (A) H-2-Kb binders. (B). H-2-Db binders.

Supplementary tables

Sup Table 1. Tiers in pVACtools. Tier available in each tool is marked with y (yes).

Tier	pVACseq	pVACsplice	pVACfuse
Pass	y	y	y
Poor Binder	y	y	y
Ref Match	y	y	y
Poor Transcript	y	y	
Low Expr	y	y	y
Anchor	y		
Subclonal	y	y	
Prob Pos	y	y	y
Poor	y	y	y
No Expr	y	y	
Low read support			y

Sup Table 2. Summary of successful case count and run time (average and median) in benchmark

pvacVer	RunParameter	Successful design	avg_runtime	median_runtime
pvacV4	default	11	70.9	52.1
pvacV5	default	11	57.5	43
pvacV4	IC1500	8	334.6	201.9
pvacV5	IC1500	8	131.2	d97.2
pvacV4	IC1500_Clip5	9	326	257.1
pvacV5	IC1500_Clip5	9	134.1	117
